# Correlation Study Between Canopy Temperature (CT) and Wheat Yield and Quality Based on Infrared Imaging Camera

**DOI:** 10.3390/plants14030411

**Published:** 2025-01-30

**Authors:** Yan Yu, Chenyang Li, Wei Shen, Li Yan, Xin Zheng, Zhixiang Yao, Shuaikang Cui, Chao Cui, Yingang Hu, Mingming Yang

**Affiliations:** College of Agronomy, Northwest A&F University, Yangling 712100, Chinashenwei1009@126.com (W.S.);

**Keywords:** infrared imaging, canopy temperature, yield, quality

## Abstract

As an important physiological indicator, wheat canopy temperature (CT) can be observed after flowering in an attempt to predict wheat yield and quality. However, the relationship between CT and wheat yield and quality is not clear. In this study, the CT, photosynthetic rate (Pn), filling rate, wheat yield, and wheat quality of 68 wheat lines were measured, in an attempt to establish a connection between CT and yield and quality and accelerate the selection of new varieties. This experiment used an infrared imaging camera to measure the CT of wheat materials planted in the field in 2022. Twenty materials with significant temperature differences were selected for planting in 2023. By comparing the temperature trends in 2022 and 2023, it is believed that materials 4 and 13 were cold-type materials, while materials 3 and 11 were warm-type materials. The main grain filling period of cold-type materials occurs in the middle and late stages of the grain filling period and the Pn and the thousand-grain weights of cold-type materials were higher than those of warm-type materials. Similarly, under continuous rainy conditions, cold-type materials had a higher protein and wet gluten contents, while warm-type materials had higher sedimentation values and shorter formation times.

## 1. Introduction

Wheat is one of the most important grain crops, and the improvement of wheat yield is critical to ensure food security globally [1]. Early wheat breeders selected high-yielding wheat varieties by measuring the weight of wheat grains, a method that was time-consuming, labor-intensive, and prone to errors caused by manual measurements [2]. Similarly, the newer methods, such as using a molecular marker method, can be relatively cumbersome [3]. Therefore, utilizing easily observable physiological indicators in combination with conventional breeding would be a promising option to increase wheat yields [4].

The physiological indicators guiding high-yield breeding of crops must be accurate, fast, repeatable, non-destructive, and low-cost [5]. Canopy temperature (CT) measurement has the advantages of being simple, affordable, and non-destructive. CT generally refers to the average temperature of the spikes, stems, and leaves on the surface of the crops. Previous studies have found that wheat with low CT can achieve higher yields than wheat with high CT under drought conditions and have confirmed that CT can be used as a selection criterion for wheat breeding [6]. The use of CT to screen for high-yielding genotypes is based on the fact that low-CT genotype wheat performs physiologically and metabolically better than high-CT genotype wheat, and low-CT genotype wheat after flowering has higher grain counts and higher yields [7]. A study by Mason and Singh [8] proved that during the breeding process, high-CT measurements can be applied to remove low-yield materials, which can greatly reduce the workload of a breeder. The cold-type wheat variety Xinong 805 recorded the highest wheat yield in Shaanxi Province China, which suggests that cold-type wheat does have higher yields [9]. This also shows that CT can be used as an important physiological index to guide wheat breeding.

When using handheld infrared thermometers to measure crop temperatures, you can often only measure values at a few points and extrapolate the crop canopy temperature from there, but infrared imaging camera can measure the temperature of the entire canopy and artificially eliminate undesirable areas at a later stage [10]. Infrared imaging camera can provide information about objects beyond visible light, such as temperature, shape and composition, and has been increasingly used in agriculture in recent years [11,12]. Saint et al. [13] used an infrared imaging camera to study the correlation between CT and grain yield, as well as differences in genotype between different varieties. Biju et al. [14] also used an infrared imaging tool to screen and identify the genotype of drought-resistant lentils. Deery et al. [15] demonstrated that CT phenotypes obtained by infrared imaging cameras were highly reproducible in the selection of genetic studies and plant breeding programs. Kunz et al. [16] found that CT had a high heritability, but its correlation with wheat yield was not significant, and it was speculated that CT may be caused by the high energy radiation from the soil and the resulting increase in plant temperature. The purpose of our experiment was to (1) use an infrared imaging camera to investigate the relationship between CT and yield; (2) identify the warm-type and cold-type of wheat; (3) investigate the relationship between CT and wheat quality. These results could expand the application potential of CT as a breeding physiological index, which may provide suitable screening tools for wheat high-yield breeding.

## 2. Results

### 2.1. Measurements CT in 2022 and 2023

**Figure 1:**
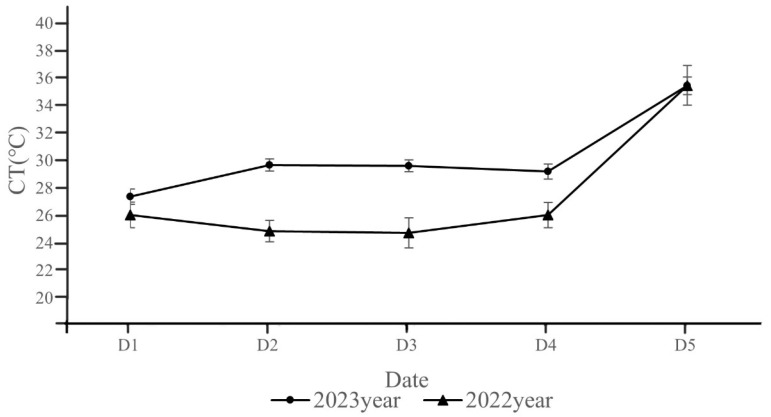
CT comparison of mid- and late stage of wheat grain filling in 2022 and 2023. D1 corresponds to 11 May 2022 and 12 May 2023; D2 corresponds to 15 May 2022 and 14 May 2023; D3 corresponds to 18 May 2022 and 18 May 2023; D4 corresponds to 21 May 2022 and 20 May 2023; D5 corresponds to 28 May 2022 and 6 June 2023. Eror bars in the figure are standard errors.

The mean CT values of all wheat materials on each measurement day were compared (Figure 1). In 2022, the CTs of wheat showed a tendency to decrease and then increase, then rise sharply at the time of dewatering at the maturity stage. In 2023, the CTs of wheat showed a tendency to increase and then level off, then rise at the time of dewatering at the maturity stage. Moreover, the CT values on each measurement day in 2023 were higher than the CT values in 2022. The differences showed a tendency of increasing and then decreasing, until the CT values of both years were basically the same at full maturity.

**Figure 2:**
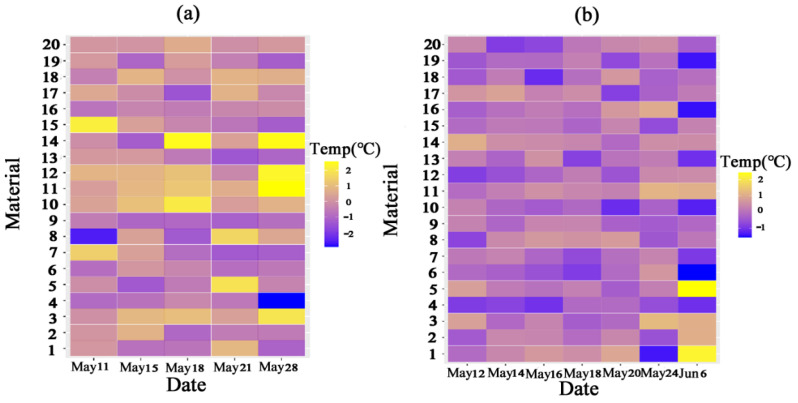
Normalized trend of CT data of wheat materials in 2022 (**a**) and 2023 (**b**). The Y-axis represents the 20 materials, and the X-axis represents the time at which the CT was measured.

CT values of different materials in 2022 and 2023 are shown in Figure 2. To eliminate the environmental changes between the two years, the values were normalized so that the relative CT values were in the range of −2~2 °C. During the wheat filling process, the differences between CTs became more significant, and some materials had different CT trends in different years. For example, material 1 showed a decreasing trend in CT in 2022 and an increasing trend in 2023; material 10, on the contrary, showed an increasing trend in 2022 and a decreasing trend in 2023. This kind of unstable temperature change was seen in what is referred to as mediate-type wheat. Most of the materials had different temperature trends in two years, but the CT of materials 4 and 13 maintained a decreasing trend over the two years, and the CT of materials 3 and 11 maintained an increasing trend. These were chosen as cold-type wheat and warm-type wheat, respectively, based on the trends and averages in the data.

Therefore, we selected warm-type wheat and cold-type wheat based on the trend and average values in the data. Taking all factors into account, it was concluded that materials 4 and 13 were cold-type wheats, while materials 3 and 11 were warm-type wheats. In the following article, we will focus on the basic characteristics of cold-type wheat and warm-type wheat through the four most contrasting materials.

Table S1 shows that in 2022, the average thousand-grain weight of cold-type wheats was about 3-11g heavier than that of warm-type wheats. However, the thousand-grain weights of materials 4 and 13 in 2023 were not significantly different from materials 3 and 11. We speculated that this may have been because the filling period of warm-type wheats was mainly in the front and middle stages, while the filling period of cold-type wheats was in the middle and particularly late stages, and required better lighting conditions.

### 2.2. Relationship Between CT and Pn

**Figure 3:**
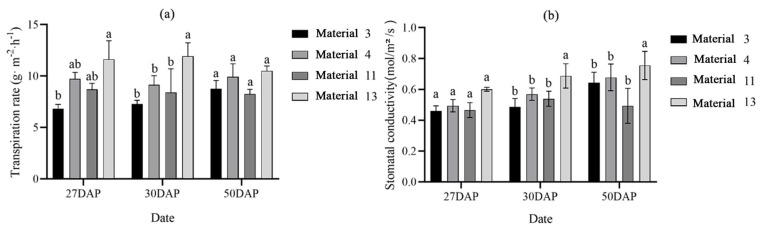
Comparison of transpiration rate and stomatal conductivity at grain filling stage between cold- and warm-type wheat. (**a**) The transpiration rate of materials 3, 4, 11 and 13. (**b**) The stomatal conductivity of materials 3, 4, 11 and 13. Different lowercase letters after the same column of data in the table indicate a significant level of up to 1% between the combined treatments. Eror bars in the figure are standard errors.

As shown in Table S2, most wheat materials showed a trend of Pn increasing and then decreasing on the three measurement dates, except for material 16 and material 19. For material 16 and 19, the Pn was higher at 27 DAP (days after flowering) than at 33 DAP, but the changes were very small—at around 1 μmol CO_2_ m^−2^ s^−1^. The Pn was low at all three measurement dates for materials 1, 3, 5, 11, 12, and 20, which also corresponded to lower stomatal conductance and transpiration rates, while materials 4, 6, and 7 had relatively high Pns. Transpiration rate analyses of the identified cold- and warm-types of wheat revealed higher transpiration rates for cold-type wheats 4 and 13 than for warm-type wheats 3 and 11. Additionally, material 13 was found to have a much higher transpiration rate than the other materials. The difference in transpiration rate between the two types was not significant at the end stage, but the transpiration rate of the cold-type wheats was still higher than that of the warm-type wheats. Further analysis of the stomatal conductance of cold- and warm-type wheats showed that the stomatal conductance of cold-type wheat increased and was higher than that of warm-type wheat with the onset of filling, and the stomatal conductance of material 11 tended to increase and then decrease (Figure 3).

**Figure 4:**
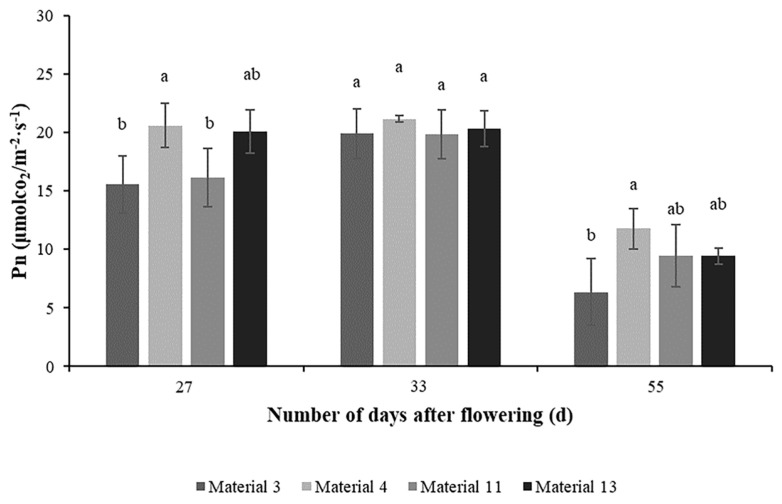
Photosynthesis in the middle and late stages of filling in 2023. Pn: net photosynthetic rate (μmol CO_2_ m^–2^ s^–1^); Different lowercase letters after the same column of data in the table indicate a significant level of up to 1% between the combined treatments. Eror bars in the figure are standard errors.

Comparison of Pns between cold-type wheat and warm-type wheat during the mid and late stages of filling (Figure 4) showed that the Pn of wheat during these stages showed a tendency to increase and then decrease, which mainly reflected the physiological changes of wheat during the growth cycle. In the middle of the filling period, the Pn peaked due to vigorous plant growth and active leaf function. With the arrival of the late filling stage, the plant gradually senesced and leaf function declined, resulting in a decrease in the Pn. The results also showed that the Pns of materials 4 and 13 were greater than those of materials 3 and 11, and that the Pns of the cold-type wheat were 20.0% and 23.07% higher than those of the warm-type wheat, respectively.

**Figure 5:**
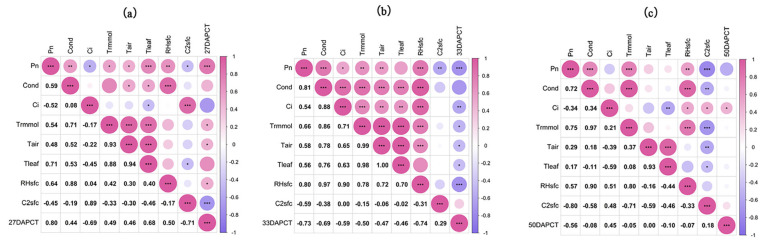
Correlation between photosynthetic parameters and canopy temperature. (**a**) Correlation between CT and photosynthetic parameters at 27 days after wheat flowering. (**b**) Correlation between CT and photosynthetic parameters at 33 days after wheat flowering. (**c**) Correlation between CT and photosynthetic parameters at 50 days after wheat flowering. *** At the 0.001 level, the correlation is significant; ** At the 0.01 level, the correlation is significant; * At the 0.05 level, the correlation is significant. Pn: net photosynthetic rate (μmol CO_2_ m^–2^ s^–1^); Cound: stomatal conductance (mol H_2_O m^–2^ s^−1^); Ci: intercellular CO_2_ concentration (μmol CO_2_ m^−1^); Trmmol: transpiration rate (mmol H_2_O m^–2^ s^–1^); Tair: air temperature (°C); Tleaf: leaf temperature (°C); RHsfc: leaf surface humidity %; C2sfc: Leaf CO_2_. concentration (μmol CO_2_ m^−1^); 27 DAPCT: CT for 27 days after flowering (°C); 33 DAPCT: CT for 33 days after flowering (°C); 50 DAPCT: CT for 50 days after flowering (°C).

Photosynthesis-related parameters for the 20 materials sown in 2023 are shown in Table S2. And Figure 5 shows the correlation between photosynthetic parameters and CT. As shown in Figure 5a, Pn was significantly positively correlated with Cond (*p* < 0.01), Trmmol (*p* < 0.05), Tair (*p* < 0.05), Tleaf (*p* < 0.001), RHsfc (*p* < 0.05), and CT (*p* < 0.001) at 27 DAP; Trmmol (*p* < 0.05), Tair (*p* < 0.05) and RHsfc (*p* < 0.05) were positively correlated with CT. This may imply that an increase in CT at 27 DAP, i.e., the filling stage, can promote photosynthesis and transpiration in plants within the appropriate temperature range. As shown in Figure 5b, Pn, Cond, Ci, Trmmol, Tair, Tleaf, and RHsfc were positively correlated with each other as the filling progressed up to 33 DAP, but the CTs at that time were negatively correlated with Pn (*p* < 0.001), Ci (*p* < 0.01), Trmmol (*p* < 0.05), Tair (*p* < 0.05), Tleaf (*p* < 0.05), and RHsfc (*p* < 0.001). This may have been due to the adverse effect of high CT on the growth of wheat, resulting in the suppression of wheat’s physiological activities. As shown in Figure 5c, at 50 DAP, CT was negatively correlated with Pn, Cond, Trmmol, Tleaf, and RHsfc, but the correlation was not significant, and CT was positively correlated with Ci (*p* < 0.05). This relationship indicated that at 50 DAP, the effects of CT on a number of physiological indexes and environmental factors were not significant, reflecting the weakening response of wheat to environmental conditions during the maturity stage and the possible adverse effects of high temperature on the physiological activities of wheat.

### 2.3. Relationship Between CT and Filling Rate

**Figure 6:**
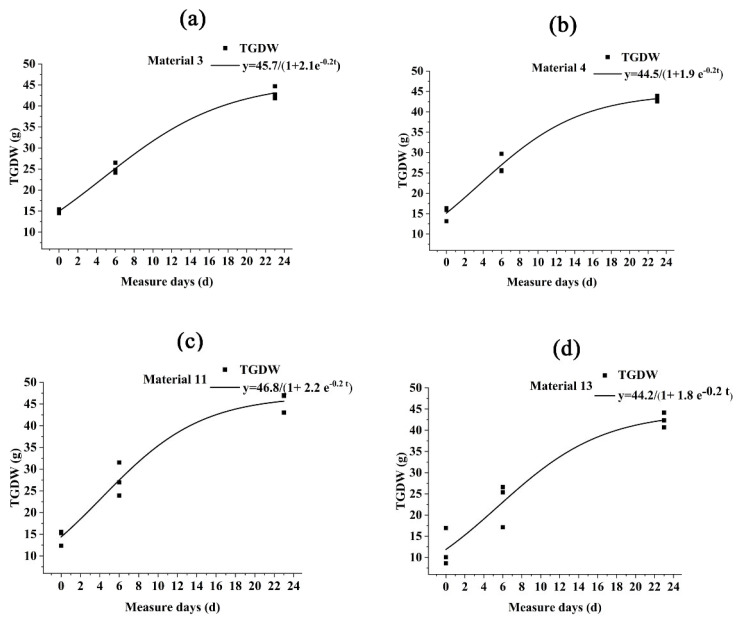
Fitting image of Logistic equation in the middle and late stages of wheat filling. (**a**) Material 3 fitting equations. (**b**) Material 4 fitting equations. (**c**) Material 11 fitting equations. (**d**) Material 13 fitting equations. TGDW: thousand dry grains weight (g).

We found a significant difference in the thousand-grain weights between cold- and warm-type wheats. We therefore explored whether there were differences in filling rates between different materials. We used the logistic growth curve to fit the grain development curve and tested its R^2^ > 0.95, indicating that the logistic equation could truly simulate the wheat grain filling process. The filling curves of materials 3, 4, 11, and 13 were arranged in sequence (Figure 6). The grain filling process in the middle and later stages of filling followed the growth pattern of “fast first and then slow”. The filling speed was faster in the middle stage and gradually stabilized in the later stage. We found that the C_0_ of warm-type wheat was higher than that of cold-type wheat, while the Vmax and T of cold-type wheat were both higher than those of warm-type wheat. This indicated that warm-type wheat had a large grain accumulation and fast filling rate in the early and middle stages of filling, while the main filling time of cold-type wheat was in the middle and late stages, with a long filling duration and a late end time. In 2023, there was continuous rainfall in the middle and late stages of wheat filling, so cold-type wheat was more affected than warm-type wheat. Therefore, we speculated that this was also the reason for the low yield of cold-type wheat in 2023.

**Figure 7:**
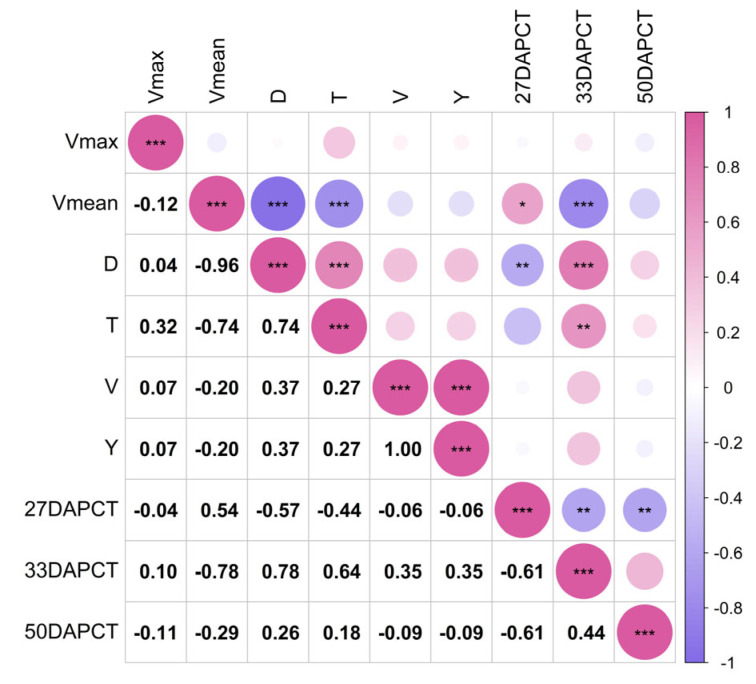
Correlation between canopy temperature and filling parameters. *** At the 0.001 level, the correlation is significant; ** At the 0.01 level, the correlation is significant; * At the 0.05 level, the correlation is significant. Vmax: the maximum grain-filling rate in the middle and late stages (g/d); Vmean: average grain filling rate in the middle and late grain-filling stages (g/d); D: active grain-filling period (d); T: grain filling end time (d); V: grain-filling velocity (g/d); Y: Late grain accumulation (g); 27 DAPCT: CT 27 days after flowering; 33 DAPCT: CT 33 days after flowering; 50 DAPCT: CT 50 days after flowering.

As shown in Figure 7, CT was significantly and positively correlated with Vmean (*p* < 0.01) and negatively correlated with D (*p* < 0.05) at 27 DAP, at the filling stage of wheat. This suggested that at a higher CT, the average filling rate of the crop would increase accordingly. This may be because suitable temperature conditions promoted the physiological activities and metabolic processes of the crop, enabling the crop to absorb and transform nutrients more effectively, thus accelerating the rate of filling and shortening the active period of growth. At 33 DAP, the wax ripening stage, the CT was significantly negatively correlated with Vmean (*p* < 0.001) and was significantly positively correlated with D (*p* < 0.001) and T (*p* < 0.01), suggesting that at that time, CT had a significant influence on the average filling rate of the crop. It indicated that the effect of CT on wheat growing at that time was opposite to the effects during the filling stage, where the average rate of crop growing was reduced at a higher CT. This may be because as the crop grew and developed into its later stage, a high temperature may have adversely affected the physiological processes of the crop, leading to the reduction of enzyme activity, metabolic disorders, and so on, ultimately slowing down the rate of filling. The higher the CT, the lower the average filling rate, the longer the active growth period, and the later the end of growing, resulting in late-maturing material that was more susceptible to hot weather. At 50 DAP, the crop may have been in the later stages of growth, approaching maturity or already ripening. At this stage, the physiological processes of the crop may have stabilized or were nearing completion, so the correlation between changes in CT and the rate and timing of filling was no longer significant.

**Figure 8:**
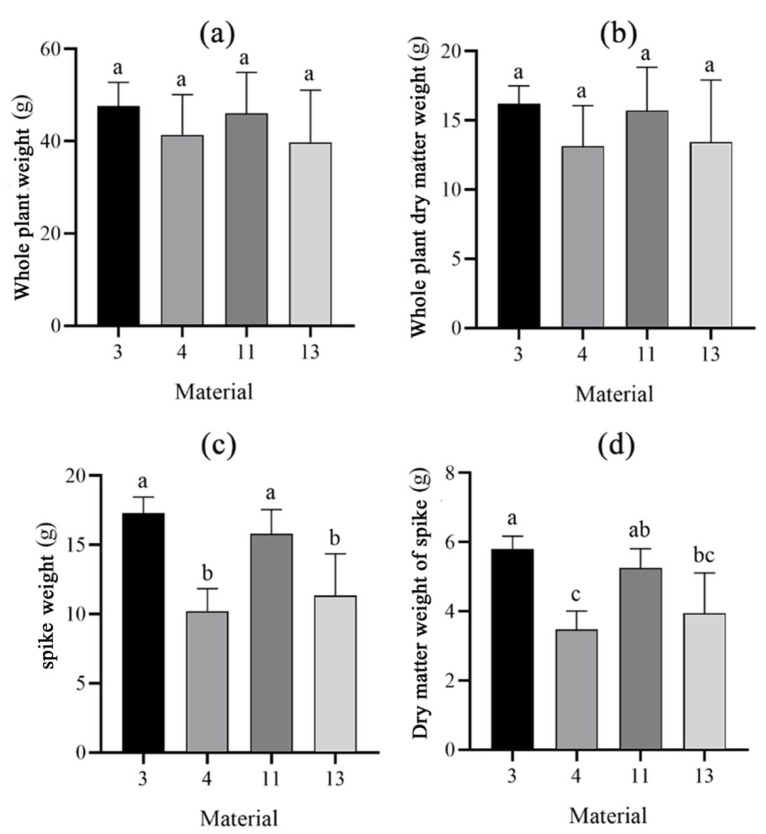
Dry matter analysis of cold- and warm-type wheat at 27 DAP. (**a**) Whole plant weight. (**b**) Whole plant dry matter weight. (**c**) Spike weight. (**d**) Dry matter weight of spike. Different lowercase letters after the same column of data in the table indicate a significant level of up to 1% between the combined treatments. Eror bars in the figure are standard errors.

**Figure 9:**
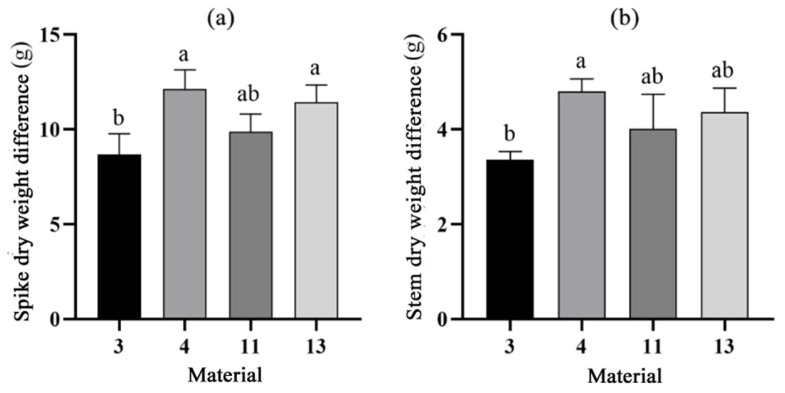
Dry weight difference in values of cold- and warm-type wheat between 33DAP and 27DAP. (**a**) Spike dry weight difference. (**b**) Stem dry weight difference. Different lowercase letters after the same column of data in the table indicate a significant level of up to 1% between the combined treatments. Eror bars in the figure are standard errors.

Next, we analyzed wheat dry matter accumulation. To explore the differences between the two types of materials, wheat spikes and stalk plants at 27 DAP and 33 DAP were measured for dry weight. It was found that there were no significant differences in the fresh weights of the single wheat plants of the four materials, and there were no significant differences between the dry weights of the whole wheat plants after drying. However, the fresh weights of individual wheat ears of materials 3 and 11 were higher than those of materials 4 and 13, with material 3 having the highest fresh weight of ears. The comparison after drying revealed that the dry weights of spikes from materials 3 and 11 were larger than those of materials 4 and 13, and were significantly different (Figure 8). To further investigate the growth differences between cold-type and warm-type wheats, the dry weight of 33 DAP was subtracted from the dry weight of 27 DAP (Figure 9). It was found that the largest difference in dry weights between the two periods was in the cold-type wheat, with materials 4 and 13 showing an increase of more than 11 g in dry weight, whereas the warm-type wheat showed a significantly smaller increase in weight. The stem dry weights were higher in the cold-type wheat than in the warm-type wheat, and the smallest difference was found in material 3 (Figure 9). This result suggested that the dry matter accumulation of warm-type wheat was higher than that of cold-type wheat until 27 DAP, and between 27 and 33 DAP, the dry matter accumulation of cold-type wheat was higher than that of warm-type wheat.

### 2.4. The Relationship Between CT and Quality

Comparison of cold-type and warm-type wheat qualities (Figure S1) revealed that cold-type wheat (materials 4 and 13) had higher GPC and WGC, and warm-type wheat (materials 3 and 11) had higher SV, DST, MR, SD, and AC. However, these differences were not significant.

**Figure 10:**
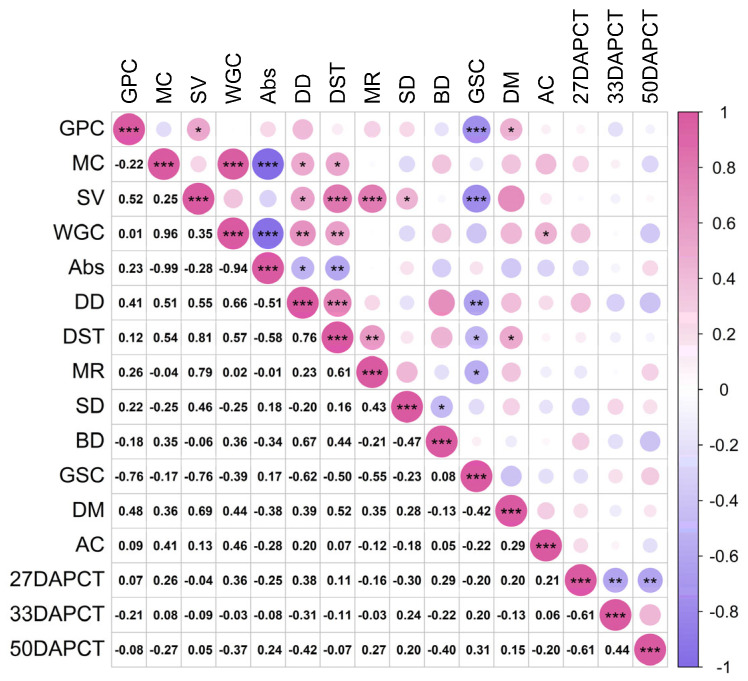
Correlation between canopy temperature and wheat quality. *** At the 0.001 level, the correlation is significant; ** At the 0.01 level, the correlation is significant; * At the 0.05 level, the correlation is significant. GPC: grain protein content (%); MC: moisture content (%); SV: sedimentation value (mL); WGC: wet gluten content (%); Abs: water absord (%); DD: dough development time (min); DST: dough stable time (min); MR: max-resistance to extension (EU); SD: degree of softening (BU); BD: bulk density (g/L); GSC: grain starch content (%); DM: ductility and malleability (mm), AC: amylose content (%); 27 DAPCT: CT for 27 days after flowering (℃); 33 DAPCT: CT for 33 days after flowering (℃); 50 DAPCT: CT for 50 days after flowering (℃).

The correlation between CT and wheat quality (Figure 10) showed that the correlation between wheat quality and CT varied after the number of days of flowering, but no significant correlation was found between the values of CT obtained by infrared imaging camera and wheat quality. This may be because the infrared imaging camera used in this experiment had a wavelength range of 7.5–14.0 µm, while the infrared range of wheat quality can be measured from 0.75–2.5 µm [17], and therefore the differences in wheat quality could not be obtained by infrared imaging camera.

## 3. Discussion

### 3.1. Relationship Between CT and Environment in Wheat

Plant canopy temperatures have been studied since 1960, with experiments exploring the optimal time to measure canopy temperatures as the afternoon. CT reproducibility is highest in the afternoon and the correlation between CT and phenotype is highest in the afternoon during the grain filling period [18]. And we did the same in our current experiment, we obtained CT of wheat with an infrared imaging camera in the afternoon during the grain filling period of wheat.

Because the dates on which wheat canopy temperatures were measured during the two growing seasons were slightly different, we recorded the adjacent dates between the two years as one point in time, so we compared a total of five points (D1–D5) (Figure 1). CT is the result of the combined effects of external environmental temperature and physiological and biochemical reactions in wheat. It is necessary to comprehensively consider changes in external environmental conditions while studying CT. Therefore, we recorded some airborne data such as atmospheric temperature and air humidity on the days we measured the CT of wheat. In addition, soil temperature is also an important environmental factor, soil temperature is determined by soil moisture content; the higher the moisture content, the lower the soil temperature. In this experiment [19], the irrigation method is natural precipitation, so the soil moisture content is regarded as the same. Moreover, this experiment had avoided photographing the bare ground when measuring the canopy temperature of wheat, so the temperature of the soil was not recorded in this experiment.

**Figure 11:**
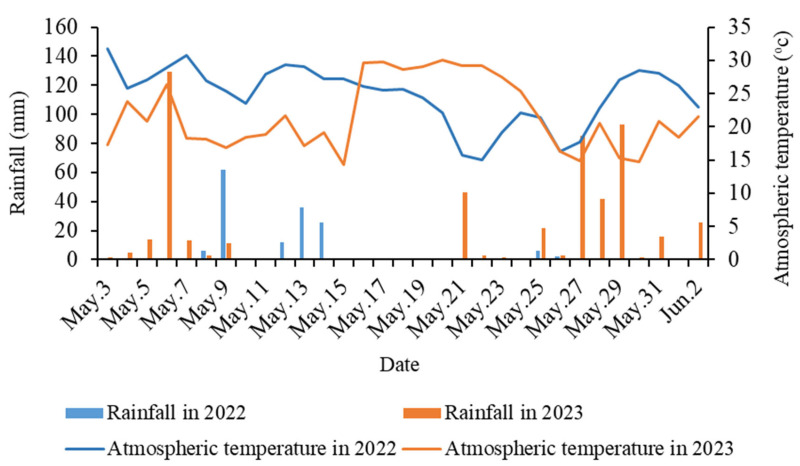
Comparison of rainfall and atmospheric temperature during wheat filling in 2022 and 2023.

In 2023, large-scale heavy rainfall occurred in the central and western regions of China, affecting 1.86 million hectare of wheat, or 8% of the national wheat area, and reducing wheat production in Shaanxi by 120 million kg. Thirteen days of continuous prolonged heavy rainfall in 2023 resulted in atmospheric temperatures that were 4–5 °C lower than those averaged in 2022 (Figure 11). Due to the high number of rainfall days in 2023, most of the measurements were taken after the rains when the solar radiation increased and the temperature rose rapidly, resulting in an increase in CT (Figure 1). In addition, after the rainfall, wheat may have adjusted its physiological processes, such as stomatal opening and closure and transpiration, to adapt to the new environmental conditions. All these factors led to differences in CT between the two years. It is also important to note that due to high rainfall, there was high moisture in the soil in 2023, which in turn increased the CT, and this correlation increased with soil depth. The CT values in both years were almost the same in the pre-harvest period because as the wheat matured, the leaves and spikes turned yellow, the filling slowed down and eventually stopped, and the wheat was no longer physiologically active. Therefore, the CTs gradually converged to the outside atmosphere temperature, and eventually became almost the same between the two years. Overall, the CT of wheat in the two years were almost the same, with lower CT in the mid- to late-filling stage (D1–D3) and a rapid increase in CT in the ripening stage (D4–D5) (Figure 1).

### 3.2. Relationship Between CT and Yield in Wheat

CT is not only a passive reflection of ambient temperature, but it is also affected by a series of physiological responses in wheat [20]. Therefore, in this experiment, more attention was paid to the differences in CT between different lines under the same weather conditions, and each measurement day was normalized to eliminate any errors caused by environmental factors and the camera. Wheat adapts to changes in external ambient temperature by regulating the opening and closing of stomata, the angle of inclination of the leaf blades, and transpiration, thus maintaining a relatively stable internal temperature. These physiological responses can have direct or indirect effects on CT. Therefore, based on the changes in CT, material 4 and material 13 were ultimately identified as cold-type wheat, material 3 and material 11 as warm-type wheat, and the rest as mediate-type wheat.

Observing and analyzing CT changes can indirectly provide an important reference for wheat planting and management. It is projected that by 2030, about 11% of the world’s wheat acreage will be exposed to average temperatures greater than 34 °C for at least 5 days during the reproductive period [21]. High temperatures cause damage to organelles and cell membranes, a reduction in the ability to remove reactive oxygen species generated by high temperatures, a reduction in the activity of antioxidant enzymes, and an acceleration in the accumulation of reactive oxygen species to accelerate wheat senescence [22,23]. In addition, high temperatures were shown to significantly fragment the leaf wax layer, exposing more chloroplastic tissue, and wheat cooled the leaves to reduce the transpiration rate while stomata were in complete or partial closure, resulting in a lower net photosynthetic rate and ribulose bisphosphate (RuBP) carboxylase activity. This reduced electron donor, acceptor performance, and RuBP activity in the PSII reaction center and lowered the stability of photosynthesis-related proteins as well as the stability of CO_2_ fixation in the Calvin cycle [24]. The low CT may imply that in response to high temperatures, wheat is able to regulate its physiological processes more efficiently, such as adjusting stomatal openings and increasing transpiration rate, thus maintaining physiological stability and reducing damage caused by high temperatures [25]. Therefore, CT should be negatively correlated with photosynthetic rate. However, the present study found that at 27 DAP the photosynthetic rates of cold-type wheats 4 and 13 were higher than those of warm-type wheats 3 and 11 (Figure 4). Additionally, the difference in photosynthetic rates between cold-type wheat and warm-type wheat at 33 DAP was not obvious, and at 50 DAP, the rate of material 4 was much higher than those of other materials. In comparison with the amount of dry matter accumulated in wheat, it was found that the cold-type wheat gained more dry weight than the warm-type wheat in the time from 27 DAP to 33 DAP (Figure 9). This suggested that cold-type wheat reduced its CT to improve its heat tolerance, which resulted in rapid dry matter accumulation. This explanation can well explain the higher thousand-grain weight of cold-type wheat than warm-type wheat. However, the rainfall in 2023 resulted in a decrease in thousand-kernel weight for almost all wheat materials, but the warm-type wheat had the least decrease in thousand grains weight and even a slight increase in thousand grains weight for material 3. We surmised that the cause of this was mainly due to the outside climate. CT was also found to be significantly positively correlated with both photosynthetic rate and growing rate in the middle of the growing period, and significantly negatively correlated in the later period (Figure 3 and Figure 7). CT was observed to have a facilitating effect in the middle stage and a hindering effect in the late stage of wheat filling. Porter and Semenov [26] used a model to show that there was a threshold effect of wheat’s response to temperature, so we inferred that there was also a threshold effect of CT on the physiological effects of wheat, and that within a certain range, an increase in CT had a positive effect on the accumulation of wheat’s thousand-kernel weight. Once this range was exceeded, CT hindered the accumulation of dry matter in wheat. We speculated that CT had the same effect as atmospheric temperature on wheat, and that a canopy critical temperature (Tc), existed. Photosynthesis in wheat was promoted when the CT was less than Tc, and photosynthesis was hindered when the CT was greater than the Tc. It has been hypothesized that this phenomenon is due to differences in the thermal sensitivity of different enzymes, changes in the rate of CO_2_ assimilation and electron transport per unit leaf area, and impaired cellular carbon metabolism, sucrose synthesis, and carbon and nitrogen partitioning within and between organs [27].

It is also possible that the reproductive period of wheat affects the correlation between CT and photosynthetic rate, with a positive correlation between CT and photosynthetic rate at milk maturity and a negative correlation at wax maturity, for reasons that need to be verified in future experiments. In addition, short-term effects involving altered gene expression, such as heat shock protein synthesis, may occur. These can lead to altered carbon effectiveness affecting the uptake, transport, and assimilation of other nutrients, while also disrupting lipid metabolism and damaging cell membranes, which are manifested in plant phenotypes as different trends in CT with temperature [28]. The effect of CT on wheat filling is negative in the entire mid- to late-period of the plant. High CT accelerates wheat senescence during actual field production, so the material has stopped irrigating before reaching the theoretical irrigating maximum. This is also consistent with the argument that the length of the filling period and the rate of grain growth are determined by the unique traits of the variety and the low temperature conditions under which the grain develops [29].

Genetic increases in pre-flowering CT were associated with reduced final plant height as found in the experiments of [30] Through our experiments, we found that the plant height of cold-type wheat was slightly reduced compared to warm-type wheat (Table S3).

### 3.3. Relationship Between CT and Quality in Wheat

To date, there have been relatively few studies on CT and wheat quality. Some researchers believe that an increase in CT increases the protein content of the grain [31]. CT mainly affects GPC by affecting gluten content. When the CT is high, the accumulation rate of wheat gluten is fast, which is conducive to the increase of total protein [32]. Some scholars believe that low CT will lead to higher GPC. Elevated CT leads to slower photosynthesis, which reduces nitrogen accumulation and grain transport. As canopy warming interferes with nitrogen transport or grain nitrogen assimilation, wheat has insufficient energy to absorb or assimilate nitrogen, resulting in limited nitrogen assimilation and therefore reduced protein production [22,23]. At the same time, high nitrogen plays a cooling role in the plant’s canopy [24]. In our work, we found higher GPC in the cold-type wheat cultivar (Figure S1). Not all wheat quality characters in the present study showed this trend, but this may be attributed to how the infrared imaging camera used had a different spectral range than the quality of wheat. The wavelength range of the infrared imaging camera used in this experiment was 7.5–14.0 µm, and the spectral range of wheat quality that can be measured is 0.75–2.5 µm [17], which means that differences in the spectral range of wheat quality could not be captured by our infrared thermal imaging method. And there was diffuse reflection of light during the detection process [33]. This affected the accuracy of the experimental data. This could also be one of the reasons why we did not detect correlations. In further studies, the characteristics of different spectral bands need to be examined in depth, and combined with appropriate detection methods to ensure the accuracy and reliability of the results when using optical techniques to detect wheat quality.

### 3.4. Infrared Camera Acquisition of CT in the Future

There have been reports of using UAVs with thermal infrared imaging cameras to measure plant temperatures in the area [34,35,36,37]. The infrared imaging camera used in this experiment could obtain richer source-sink information and higher image resolution compared with low-altitude UAV technology, which is a significant advantage of close-range sensing technology [38]. However, this method is still affected by external factors. Constant external changes (e.g., sun exposure angle, ambient temperature, cloud cover, etc.) make it difficult to compare measurements even over short time intervals [39,40]. Therefore, in the future, it is extremely necessary to reduce the potential errors caused by environmental instability. Some information must be discarded to obtain temperature information faster; for example, the experimental time can be reduced by using drones, and drones can be equipped with more band equipment in addition to infrared imagers to obtain more comprehensive canopy data.

## 4. Materials and Methods

### 4.1. Test Ground and Experimental Materials

Field trials were carried out in two consecutive growing seasons in 2021–2022 and 2022–2023, in the north campus of Northwest A&F University in Yangling, China (34°17′25.836″ N and 108°4′40.4904″ E). Sixty-eight advanced breeding lines were used in this experiment, and were numbered materials 1–68. In the second year, 20 wheat lines with different CT properties were screened out of the original 68 for further field experiments. In the first growing season, wheat was sown on 18 October 2021 and harvested on 2 June 2022; in the second growing season, wheat was sown on 15 October 2022 and harvested on 12 October 2023. The average altitude of the cultivation site was 530 m, the average annual temperature was 12.9 °C, and the average annual rainfall was 637.6 mm. Wheat materials were sowed in early October each year and harvested in the following June. The test was designed with a random zone group with three repetitions. The area of the community was 3 m^2^ (1.5 m × 2.0 m), and 25 cm between rows. And Xiaoyan 22 was sown as a protected row at the edge of the test land. Compound fertilizer was applied at 700 kg/ha (N:P:K = 18:18:5) before planting. Wheat growth mainly depended on natural precipitation, and field management was the same, as with local production.

Winter wheat has a long growing period and complex climatic reactions, with temperature and rainfall being the two climatic factors that cause the greatest variability in yields [41]. Therefore, while measuring CT, we collected rainfall and atmospheric temperature for two years (Rainfall and atmospheric temperature are both available at https://rp5.ru/) (Accessed on 15 September 2023) (Figure 11). In 2023, the rainfall during the wheat filling period was 539 mm, exceeding the same period in 2022 by 143%. In 2022, the rainfall was mainly distributed in the pre-filling and middle periods. In 2023, rainfall was high throughout the entire period of wheat filling, especially in May during the latter part of the filling period. That month, there was 13 days of continuous prolonged rainfall, with a continuous cumulative rainfall of 339.3 mm, which accounted for 63% of the total rainfall in the filling period. The high rainfall in 2023 resulted in generally lower atmospheric temperatures than in the same period in 2022 during the wheat filling period, except from 16 May to 26 May 2023.

### 4.2. CT Measurement and Data Normalization

During the wheat filling period, an FLIR A655SC infrared imaging camera (wavelength 7.5–14.0 µm) (A655sc, FLIR Systems Inc., Wilsonville, OR, USA) was connected to a laptop computer to control image acquisition. Observations were made with the lens placed about 2 m above ground level with the lens angled about 45° downward, during the observation period from 13:00 to 14:00, when the differences in CT between different wheat materials were most pronounced [39]. Photographs were taken to avoid the effects of bare ground and repeated three times. Wheat canopy temperatures can only be measured using infrared imaging on clear, cloudless days, unsuitable weather was encountered and the measurement dates needed to be postponed, resulting in the same number of measurements in both years. The measurement dates for 2022 were 11 May, 15 May, 18 May, 21 May, and 28 May, and for 2023 were 12 May, 14 May, 18 May, 20 May, 24 May, 28 May and 6 June.

After the infrared photographs were acquired, the photographs were imported into ResearchIR, the photo processing software accompanying the infrared imaging camera. The atmospheric conditions at the time of the photographs were entered, including atmospheric temperature, atmospheric humidity, and refractive index of light, for correction of the resulting photographs. The appropriate size of ROI was then selected to circumvent the undesirable areas of the photographs, and finally the wheat CT data were obtained. The Ohnishi et al. [39] and Niedz et al. [42] methods were referred to for normalizing CT data of the same row of material to eliminate the effects of differing external environments during measurement. This was performed by subtracting the average of the neighboring rows. In this method, the normalized CT was calculated by subtracting the adjacent line (in this experiment, the adjacent line was the average of the six materials adjacent to the target material) from the normalized target. The formula was [CT value of the target] − [average value of the neighboring lines around the target].

### 4.3. Filling Rate and the Accumulation of Material on the Ground Measurement

A total of 100 spikes with the same date of flowering and uniform growth were selected and labeled. Samples were taken from the field after obtaining temperature images, and 5 spikes were collected for each material each time. The samples were held at 105 °C for 10 min, and then at 80 °C until they reached a constant weight. Logistic model statistics were used to fit the variation of the filling rate of each material, and the logistic equations are shown below [43]:

The grain growth process was simulated using the logistic equation:

y = k/(1 + a e^−b T^) (1)

where y was the weight of a thousand grains, t was the number of days (t_0_ on the first day of sampling, that is, 27 days after flowering), k was the theoretical maximum thousand-grain weight, and a and b were coefficients to be determined.

The following filling rate equation was derived:

V(t) = k a b e^−b T^/(1 + ae^−b T^)^2^ (2)

Let t = 0 in Equation (1) to measure the initial grain weight C_0_ (g):

C_0_ = k/(1 + a) (3)

Substituting Equation (2) yielded the maximum filling rate of a thousand grains; Vmax (g/d):

Vmax = kb/4(4)

The average filling rate of the filling process; Vmean (g/d):

Vmean = (kb)/6(5)

It was assumed that when y reached y_0_ 96%, it represented the actual filling period, T, which was substituted into Equation (1) to obtain the filling end time:

T = −[ln (100/96 − 1)/a]/b(6)

T was substituted into (1) and (2) to obtain the late filling rate, V (g/d), and the late grain accumulation, Y (g).

On the day of CT measurement, three wheat plants from each material were collected and brought back to the laboratory, and the fresh weights were measured. Then the plants were placed in an oven at 105 °C for 30 min, dried at 80 °C until a constant weight was reached, and then measured to find the dry matter weight of the wheat plants.

### 4.4. Photosynthetic Assay

Photosynthetic parameter determination and temperature measurement were performed on the same day (10:00–12:00) [44,45]. Photosynthetic parameters were measured using an LI-COR 6400 photosynthesis assay system (LI-COR Inc., Lincoln, NE, USA). The light intensity in the leaf chamber of the photosynthetic system was set to 1000 μmol m^−2^ s^−1^.

### 4.5. Analysis on Agronomic Traits

The plant height (PH) were measured in the field. As a control, ten spikes were randomly selected, and the measurements were repeated three times. Each material was harvested on 2 June, after fifty spikes from each material were randomly selected at harvest to be threshed by a thresher, and grain weights were measured using an electronic balance.

### 4.6. Measurement of Wheat Quality

Fifty spikes of each material were randomly selected after harvest. A near infrared analyzer (DA7250, Perten, Hägersten, Sweden) was used to measure grain quality values. A near infrared analyzer provided a fast, non-destructive and environmentally friendly method of analysis [46]. Quality indicators including grain protein content (GPC), moisture content (MC), sedimentation value (SV), wet gluten content (WGC), water absorption (Abs), dough development time (DD), dough stable time (DST), max-resistance to extension (MR), degree of softening (SD), bulk density (BD), grain starch content (GSC), ductility and malleability (DM), and amylose content (AC) [47]. Each sample was evaluated in triplicate.

### 4.7. Data Processing

Data were collected and collated using Excel 2019. Curve fitting of the growing equations was carried out using an Origin 2022 software Slogistic3 model and the variation in grouting rate for each material was fitted using logistic model statistics. Correlation analysis was performed using IBM SPSS Statistics 25.

## 5. Conclusions

In this work, we used an infrared imaging camera to distinguish canopy temperature types (CT) in wheat. And the effects of different canopy temperature types on photosynthesis and irrigation rate of wheat were revealed by the infrared imaging camera. The usability of the infrared imaging camera in the breeding process was demonstrated. The study also found that under favorable environmental conditions, cold-type wheat had higher thousand-grain weights than warm-type wheat, while during excessive rainfall, thousand-grain weights of warm-type wheat showed less decline than the weights of cold-type wheat. Therefore, in wheat breeding, CT data obtained from infrared imaging camera can provide valuable references for breeders, especially when considering planting areas under different external conditions. Correlations between wheat CT and wheat quality are shown to exist, but extensive experimental evidence is still lacking. Further study is necessary to continue exploring this relationship.

## Data Availability

The original contributions presented in this study are included in the article/Supplementary Material. Further inquiries can be directed to the corresponding authors.

## Supplementary Materials

The following supporting information can be downloaded at: https://www.mdpi.com/article/10.3390/plants14030411/s1, Table S1. Different materials 2022 and 2023 thousand grain weight. Table S2. Photosynthetically related parameters of 2023. Table S3. Flowering dates and plant height of cold- and warm-style materials in 2023. Figure S1. Comparison of grain filling quality of cold- and warm-style wheat.
