# Supplementary material for: Correlation Study Between Canopy Temperature (CT) and Wheat Yield and Quality Based on Infrared Imaging Camera
Source: Plants (Basel). 2025 Jan 30;14(3):411. doi: 10.3390/plants14030411 (PMC11820293; doi:10.3390/plants14030411)
Supplement: Supplementary file 1 [file plants-14-00411-s001.zip › plants-3422187-supplementary.pdf]

Correlation Study Between Canopy Temperature (CT) and Wheat Yield and Quality Based on Infrared Imaging Camera

Yan Yu, Chenyang Li, Wei Shen, Li Yan, Xin Zheng, Zhixiang Yao, Shuaikang Cui, Chao Cui, Yingang Hu * and Mingming Yang *

Supplemental information

Table S1. Different materials 2022 and 2023 thousand grain weight.

	1	2	3	4	5	6	7	8	9	10
2022 TWG	44.95	45.06	40.98	47.43	47.22	47.58	43.67	44.35	42.04	44.38
2023 TWG	37.80	38.96	41.34	41.47	41.19	40.80	39.87	40.36	37.60	37.72
	11	12	13	14	15	16	17	18	19	20
2022 TWG	44.36	51.66	51.70	49.51	40.65	46.15	49.60	44.42	46.95	47.67
2023 TWG	42.11	34.18	39.36	38.85	42.07	40.48	42.38	41.19	41.21	34.17

Table S2. Photosynthetically related parameters of 2023.

Material	Day (d)	Pn (μmol CO_2 $m^{-2}s^{-1}$)	Cound (mol H_2O $m^{-2}s^{-1}$)	Ci(μmol CO_2 m^{-1})	Trmmol (mmol $H_2Om^{-2}s^{-1}$)	Tair (°C)	Tleaf (°C)	RHsfc (%)	C2sfc (μmol CO_2 m^{-1})
	27DAP	15.18±2.15a	0.53±0.07a	313.05±26.31a	10.84±4.64a	34.70±5.55a	32.32±4.61a	62.09±6.58a	372.93±26.76a
1	33DAP	19.61±1.71a	0.56±0.09a	319.21±8.97a	7.20±2.13a	28.43±3.80a	27.80±3.63a	68.66±2.31a	384.90±5.84a
	50DAP	7.99±1.07a	0.31±0.10a	379.98±11.03a	7.40±3.06a	32.84±3.07a	31.99±2.59a	53.61±3.58a	442.63±17.72a
	27DAP	17.18±1.97a	0.67±0.15a	319.87±29.12a	8.89±1.20a	31.49±1.13a	29.85±1.53a	69.42±4.27a	371.63±30.37a
2	33DAP	20.32±1.62a	0.68±0.13a	323.51±9.13a	7.79±1.75a	28.78±3.02a	27.85±2.91a	71.31±3.55a	380.16±3.11a
	50DAP	11.84±3.38a	0.37±0.17a	357.00±18.47a	9.25±2.59a	34.36±1.03a	33.68±1.05a	50.72±9.22a	432.12±8.28a
	27DAP	15.55±2.86a	0.55±0.09a	337.80±16.62a	8.08±1.50a	31.28±1.61a	29.19±1.86a	65.96±2.75a	392.93±14.78a
3	33DAP	19.90±1.87a	0.46±0.03a	316.73±7.05a	6.84±0.41a	27.23±0.96a	26.43±0.91a	68.40±1.44a	378.90±2.29a
	50DAP	6.66±2.70a	0.49±0.05a	361.75±29.44a	7.28±0.38a	33.25±1.83a	33.00±1.85a	44.65±4.16a	448.34±20.88a
	27DAP	20.62±2.19a	0.64±0.07a	305.47±9.08a	8.80±0.79a	31.71±1.84a	31.00±1.58a	65.89±2.76a	382.32±4.96a
4	33DAP	21.17±0.60a	0.49±0.04a	311.21±7.84a	9.73±0.63a	26.84±0.63a	25.85±0.98a	68.94±2.52a	376.26±3.53a
	50DAP	11.69±2.98a	0.57±0.04a	361.30±21.08a	9.16±0.88a	33.07±2.66a	32.44±2.41a	52.94±5.69a	439.38±15.62a
	27DAP	14.04±2.64a	0.68±0.09a	340.10±21.41a	9.93±1.28a	31.30±1.32a	29.19±1.70a	64.85±2.79a	396.09±13.44a
5	33DAP	17.84±2.46a	0.45±0.06a	310.13±8.89a	6.04±0.59a	27.37±0.56a	26.68±0.72a	63.39±2.37a	383.55±2.93a
	50DAP	7.58±2.99a	0.23±0.10a	364.19±15.06a	6.58±2.80a	33.84±1.82a	33.25±1.48a	47.88±3.28a	440.77±16.81a

Different lowercase letters after the same column of data in the table indicate a significant level of up to 1% between the combined treatments. Pn: net photosynthetic rate (μmol CO_2 m^{-2} s^{-1});Cound:stomatal conductance (mol H_2O m^{-2} s^{-1});Ci: intercellular CO_2 concentration (μmol CO_2m^{-1}); Trmmol: transpiration rate (mmol H2O m^{-2} s^{-1});Tair: air temperature (℃); Tleaf: leaf temperature (℃); RHsfc: leaf surface humidity % ; C2sfc: Leaf CO_2. concentration (μmol CO_2 m^{-1}); 27 DAP: 27 days after flowering (℃); 33 DAP: 33 days after flowering (℃); 50 DAP: 50 days after flowering (℃).

Table S2. Photosynthetically related parameters of 2023. (Continued)

Material	Day (d)	Pn (μmol CO_2 $m^{-2}s^{-1}$)	Cound (mol H_2O $m^{-2}s^{-1}$)	Ci(μmol CO_2 m^{-1})	Trmmol (mmol $H_2Om^{-2}s^{-1}$)	Tair (°C)	Tleaf (°C)	RHsfc (%)	C2sfc (μmol CO_2 m^{-1})
	27DAP	19.89±2.04a	0.65±0.09a	321.87±8.13a	10.20±0.80a	34.26±0.58a	33.16±0.60a	70.57±2.51a	382.59±4.98a
6	33DAP	21.08±5.83a	0.69±0.21a	319.92±96.89a	10.57±3.36a	34.28±10.45a	33.2±10.06a	71.64±21.45a	379.67±118.47a
	50DAP	11.88±2.48a	0.35±0.07a	364.62±13.46a	8.56±2.07a	33.43±1.67a	32.77±1.52a	53.89±2.45a	436.69±14.35a
	27DAP	20.73±1.91a	0.61±0.10a	315.69±8.05a	9.86±0.61a	32.93±0.58a	31.86±0.64a	67.48±3.42a	381.71±2.60a
7	33DAP	21.50±1.63a	0.72±0.09a	322.44±7.18a	10.10±0.70a	33.08±0.44a	32.13±0.56a	72.48±2.00a	380.38±1.82a
	50DAP	11.01±3.17a	0.29±0.10a	354.99±14.63a	7.53±2.48a	33.83±1.63a	33.21±1.74a	50.66±4.86a	438.74±10.70a
	27DAP	19.15±1.88a	0.65±0.09a	306.65±22.19a	10.62±1.39a	33.48±1.55a	32.15±1.17a	67.58±5.07a	365.39±22.90a
8	33DAP	20.25±1.85a	0.60±0.19a	311.50±16.75a	8.26±2.07a	30.42±2.63a	29.70±2.48a	67.78±6.90a	379.85±2.75a
	50DAP	9.59±4.55a	0.28±0.11a	367.60±12.68a	7.81±2.38a	34.27±1.34a	33.63±1.27a	48.43±6.09a	439.65±10.29a
	27DAP	19.37±2.81a	0.66±0.10a	303.47±14.10a	9.99±0.81a	33.2±1.12a	32.12±1.06a	69.83±4.00a	361.53±23.22a
9	33DAP	21.31±1.26a	0.75±0.04a	323.93±8.26a	10.23±0.33a	33.62±0.67a	32.36±0.68a	73.71±1.06a	378.97±4.13a
	50DAP	10.60±2.44a	0.29±0.08a	362.86±21.61a	7.51±1.50a	33.52±1.28a	32.92±1.26a	50.75±6.05a	441.08±10.81a
	27DAP	19.47±2.12a	0.52±0.14a	290.58±16.36a	8.57±1.20a	32.20±0.84a	31.15±0.98a	64.32±6.05a	365.71±24.04a
10	33DAP	22.22±1.23a	0.74±0.08a	320.06±3.96a	10.02±0.65a	33.16±0.69a	32.06±0.70a	73.38±1.66a	377.68±2.26a
	50DAP	10.41±3.22a	0.22±0.07a	345.65±22.92a	6.09±1.19a	33.51±1.67a	33.19±1.76a	46.40±6.47a	441.62±10.92a

Different lowercase letters after the same column of data in the table indicate a significant level of up to 1% between the combined treatments. Pn: net photosynthetic rate (μmol CO_2 m^{-2} s^{-1});Cound:stomatal conductance (mol H_2O m^{-2} s^{-1});Ci: intercellular CO_2 concentration (μmol CO_2m^{-1}); Trmmol: transpiration rate (mmol H2O m^{-2} s^{-1});Tair: air temperature (℃); Tleaf: leaf temperature (℃); RHsfc: leaf surface humidity % ; C2sfc: Leaf CO_2. concentration (μmol CO_2 m^{-1}); 27 DAP: 27 days after flowering (℃); 33 DAP: 33 days after flowering (℃); 50 DAP: 50 days after flowering (℃).

Table S2. Photosynthetically related parameters of 2023. (Continued)

Material	Day (d)	Pn (μmol CO_2 $m^{-2}s^{-1}$)	Cound (mol H_2O $m^{-2}s^{-1}$)	Ci (μmol CO_2 m^{-1})	Trmmol (mmol $H_2Om^{-2}s^{-1}$)	Tair (℃)	Tleaf (℃)	RHsfc (%)	C2sfc (μmol CO_2 m^{-1})
11	27DAP	16.15±2.24a	0.56±0.10a	333.36±24.61a	9.05±1.69a	32.21±1.92a	30.29±2.29a	64.36±3.81a	392.82±13.94a
	33DAP	19.87±2.26a	0.47±0.05a	309.27±9.54a	8.71±0.59a	27.34±1.27a	26.57±1.26a	66.18±2.86a	379.42±4.36a
	50DAP	9.62±2.52a	0.54±0.05a	368.88±27.20a	8.40±2.31a	33.70±2.17a	32.98±2.22a	50.55±9.56a	440.61±10.04a
12	27DAP	15.87±2.08a	0.49±0.11a	333.84±13.71a	8.27±0.45a	31.31±1.26a	29.28±1.59a	64.92±3.16a	393.8±11.14a
	33DAP	20.47±2.65a	0.51±0.06a	307.42±5.01a	6.17±0.33a	26.89±0.85a	26.09±1.05a	65.84±2.24a	380.09±4.02a
	50DAP	7.28±3.05a	0.25±0.10a	378.19±14.15a	6.85±2.81a	33.75±1.34a	33.04±1.23a	48.29±4.87a	445.07±10.59a
13	27DAP	20.09±5.90a	0.60±0.01a	320.31±96.45a	11.63±1.82a	34.66±10.52a	33.08±9.90a	69.01±21.08a	378.82±115.57a
	33DAP	20.35±1.66a	0.69±0.08a	312.32±11.86a	11.94±1.31a	31.63±3.32a	30.80±3.070a	67.92±5.58a	380.45±2.27a
	50DAP	9.53±2.61a	0.76±0.09a	347.95±20.54a	10.51±0.49a	33.66±0.95a	33.49±1.06a	46.80±4.67a	439.75±10.70a
14	27DAP	16.04±1.24a	0.59±0.11a	334.83±18.74a	9.31±1.16a	32.27±1.60a	30.47±2.09a	65.53±4.37a	390.95±9.20a
	33DAP	19.59±2.95a	0.52±0.08a	309.78±14.92a	6.42±0.50a	27.09±0.65a	26.37±0.69a	65.66±3.02a	380.22±1.93a
	50DAP	11.25±2.72a	0.34±0.08a	360.68±22.16a	8.79±1.74a	34.15±1.68a	33.45±1.58a	51.78±4.53a	433.13±13.61a
15	27DAP	19.09±1.44a	0.55±0.09a	299.21±18.38a	8.82±1.34a	32.87±1.64a	31.99±1.77a	68.04±2.62a	366.62±25.29a
	33DAP	20.49±5.52a	0.65±0.20a	318.03±95.04a	10.66±3.42a	34.38±10.46a	33.46±10.16a	69.56±20.86a	381.58±119.18a
	50DAP	8.87±1.76a	0.21±0.07a	343.65±28.54a	6.47±1.63a	34.80±1.97a	34.31±2.07a	45.09±6.32a	438.78±13.99a

Different lowercase letters after the same column of data in the table indicate a significant level of up to 1% between the combined treatments. Pn: net photosynthetic rate (μmol CO_2 m^{-2} s^{-1});Cound:stomatal conductance (mol H_2O m^{-2} s^{-1});Ci: intercellular CO_2 concentration (μmol CO_2m^{-1}); Trmmol: transpiration rate (mmol H2O m^{-2} s^{-1});Tair: air temperature (℃); Tleaf: leaf temperature (℃); RHsfc: leaf surface humidity % ; C2sfc: Leaf CO_2. concentration (μmol CO_2 m^{-1}); 27 DAP: 27 days after flowering (℃); 33 DAP: 33 days after flowering (℃); 50 DAP: 50 days after flowering (℃).

Table S2. Photosynthetically related parameters of 2023. (Continued)

Materia l	Day (d)	Pn (μmol CO_2 $m^{-2}s^{-1)}$	Cound (mol H_2O $m^{-2}s^{-1}$)	Ci (μmol CO_2 m^{-1})	Trmmol (mmol $H_2Om^{-2}s^{-1)}$	Tair (℃)	Tleaf (℃)	RHsfc (%)	C2sfc (μmol CO_2 m^{-1})
16	27DAP	20.94±1.74a	0.66±0.13a	315.84±7.10a	10.58±0.78a	33.94±1.39a	32.83±1.48a	69.11±3.37a	379.01±3.35a
	33DAP	19.98±1.55a	0.59±0.15a	310.70±14.32a	9.39±2.22a	32.51±3.38a	31.60±3.17a	67.18±4.62a	379.21±2.68a
	50DAP	12.38±2.75a	0.33±0.12a	345.49±23.27a	8.46±2.44a	34.41±1.65a	33.67±1.55a	51.17±7.00a	430.15±14.92a
17	27DAP	19.24±1.60a	0.62±0.10a	306.16±18.53a	9.58±0.60a	32.13±0.52a	31.06±0.48a	67.23±3.30a	367.20±25.43a
	33DAP	20.92±1.29a	0.68±0.09a	322.46±7.57a	9.70±0.51a	32.96±0.29a	32.14±0.30a	72.01±2.23a	382.10±3.67a
	50DAP	10.59±2.25a	0.32±0.06a	367.58±24.07a	8.27±1.16a	33.73±2.11a	33.05±2.02a	51.30±6.10a	439.46±12.74a
18	27DAP	17.01±2.32a	0.57±0.08a	317.98±32.80a	7.65±1.30a	30.52±1.87a	28.94±1.98a	68.41±3.34a	375.13±30.07a
	33DAP	19.33±2.19a	0.57±0.14a	314.16±15.04a	7.10±2.28a	28.78±3.56a	28.02±3.21a	69.32±4.04a	380.51±3.13a
	50DAP	11.18±3.31a	0.28±0.15a	341.21±21.84a	7.73±3.31a	34.14±2.55a	33.56±2.18a	48.03±6.37a	430.70±20.18a
19	27DAP	20.96±1.65a	0.79±0.14a	325.52±6.76a	10.93±0.77a	34.25±0.56a	32.85±0.51a	73.48±3.33a	379.11±3.07a
	33DAP	20.81±1.62a	0.74±0.14a	323.53±6.75a	10.97±0.91a	34.63±0.19a	33.18±0.27a	72.20±3.32a	380.14±2.68a
	50DAP	10.95±3.85a	0.36±0.13a	369.46±8.19a	8.76±2.61a	34.13±1.40a	33.27±1.16a	53.56±6.55a	438.47±10.51a
20	27DAP	16.86±2.21a	0.48±0.09a	298.20±19.87a	8.71±0.56a	32.68±0.45a	31.53±0.46a	62.59±4.95a	366.92±27.48a
	33DAP	19.63±1.25a	0.56±0.14a	314.17±20.01a	7.86±1.91a	30.65±2.84a	29.85±2.59a	67.98±4.91a	384.17±5.92a
	50DAP	8.95±3.30a	0.21±0.09a	348.38±27.31a	6.22±2.71a	33.36±2.85a	32.85±2.58a	45.36±5.28a	440.01±15.87a

Different lowercase letters after the same column of data in the table indicate a significant level of up to 1% between the combined treatments. Pn: net photosynthetic rate (μmol CO_2 m^{-2} s^{-1});Cound:stomatal conductance (mol H_2O m^{-2} s^{-1});Ci: intercellular CO_2 concentration (μmol CO_2m^{-1}); Trmmol: transpiration rate (mmol H2O m^{-2} s^{-1});Tair: air temperature (℃); Tleaf: leaf temperature (℃); RHsfc: leaf surface humidity % ; C2sfc: Leaf CO_2. concentration (μmol CO_2 m^{-1}); 27 DAP: 27 days after flowering (℃); 33 DAP: 33 days after flowering (℃); 50 DAP: 50 days after flowering (℃).

Table S3. Flowering dates and plant height of cold- and warm-style materials in 2023

Material	Flowering dates	Plant height(cm)	TGDW of 2023(g)
3	2023.4.25	81.85±3.10	41.34±3.49
4	2023.4.28	97.19±5.87	41.47±1.71
11	2023.4.25	82.77±5.39	42.11±3.38
13	2023.4.26	82.16±3.96	39.36±1.30

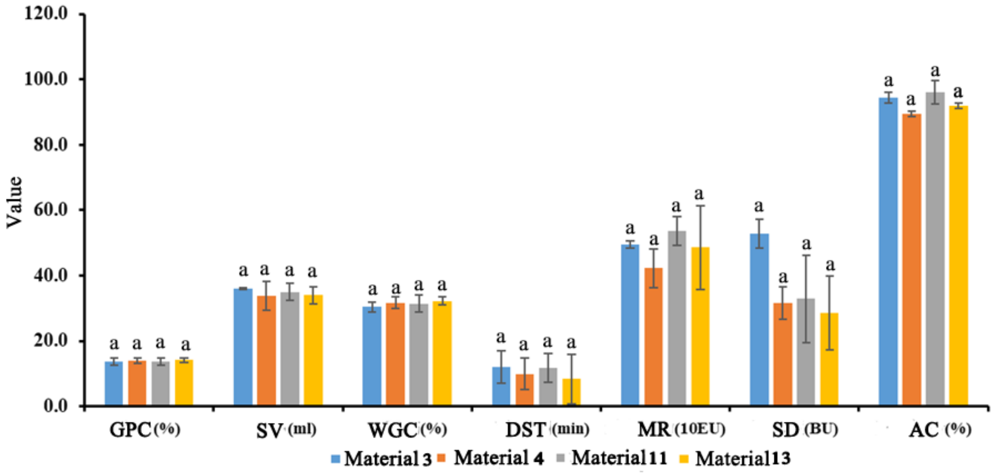

Figure S1. Comparison of grain filling quality of cold- and warm-style wheat

GPC: Grain protein content (%); SV: Sedimentation value (ml); WGC: Wet gluten content (%); DST: Dough stable time (min); MR: Max-resistance to extension (EU); SD: Degree of softening (BU); AC: Amylose content (%).

Different lowercase letters in the figure indicate a significant level of up to 5% between the combined treatments. Eror bars in the figure are standard errors.
